# The Effect of Lupus on Pregnancy and the Foetus: Should we really be Worried? A Single-Centre Retrospective Study

**DOI:** 10.31138/mjr.150923.swr

**Published:** 2024-09-30

**Authors:** Emre Kaan Cadir, Nazife Sule Yasar Bilge, Muzaffer Bilgin, Timucin Kasifoglu

**Affiliations:** 1 Department of Internal Medicine, Eskisehir Osmangazi University, Eskisehir, Turkey; 2 Division of Rheumatology, Department of Internal Medicine, Eskisehir Osmangazi University, Eskisehir, Turkey; 3 Department of Biostatistics, Eskisehir Osmangazi University, Eskisehir, Turkey

**Keywords:** systemic lupus erythematosus, antiphospholipid antibody syndrome, pregnancy

## Abstract

**Objective/Aim:**

Systemic Lupus Erythematosus (SLE), is common in women of childbearing age and is associated with obstetric complications. The aim of this study was to evaluate the course of pregnancy and its results in SLE patients with a history of pregnancy.

**Methods:**

Pregnant patients with SLE who applied to the Rheumatology outpatient clinic of between 2010 and 2020 were retrospectively screened.

**Results:**

Fifty-five pregnancies of 31 SLE patients were included in the study. Spontaneous abortion was observed in 27.3% (n:15) and foetal loss in 18.2% (n:10). Neonatal loss or maternal death was not observed in any of the patients. The rate of patients with renal involvement was 34.5% (n:19), and the rate of exacerbation was higher in pregnant women with kidney involvement (26% vs 0% (p:0.006)). Antiphospholipid antibody syndrome (APS) was present in 32.7% (n:18) of the cases, and there was a history of foetal loss before diagnosis in 50% (n:9) of the cases with APS. The foetal loss was seen only in pregnant women with APS (55.6% (n:10) vs. 0% (n:0) (p<0.001)). Six (10.9%) of 55 pregnancies in our study were unplanned pregnancies. Of these, five resulted in abortion. Exacerbation was observed in 3 of the unplanned pregnancies.

**Conclusion:**

Pregnancy complications were significantly lower in patients who were in remission since contraception. Exacerbations were more common in unplanned pregnancies and in patients with renal involvement. The presence of APS was associated with increased pregnancy morbidity.

## INTRODUCTION

Systemic lupus erythematosus (SLE) is a chronic, multisystemic, autoimmune connective tissue disease. The aetiology of SLE is still unknown. SLE most commonly affects females between the ages of 20 and 40.^1^ As a result of its frequent occurrence in women of childbearing age, SLE is associated with obstetric complications such as spontaneous abortion, preterm birth, intrauterine growth retardation (IUGR), foetal loss, preeclampsia, and increased maternal morbidity and mortality.^2^ Anti-SS-A and anti-SS-B antibodies may increase the risk of pregnancy morbidities such as congenital heart block and neonatal lupus.^3,4^ In recent studies, pregnancies without any complications have been reported in SLE patients, especially in planned pregnancies after 4–6 months of remission.^5–11^ Unplanned pregnancies, especially when the disease is not in remission, increase the likelihood of exacerbation.^3,10^ Hypocomplementemia, renal involvement, presence of anti-SS-A, anti-SS-B and antiphospholipid antibodies, and a history of foetal loss are known risk factors for obstetric complications.^12^ In this study, we aimed to evaluate the pregnancy course and outcomes of SLE patients followed by a tertiary centre rheumatology clinic.

## MATERIALS AND METHODS

In this study, we evaluated the pregnant SLE patients who applied to the rheumatology outpatient clinic between 2010 and 2020. Demographic data, obstetric history, organ involvement before or during pregnancy, SLE exacerbation during pregnancy, laboratory findings such as complete blood count at the last pre-pregnancy visit, liver function tests, serum creatinine, and complement levels, urinalysis, autoantibody tests including ENA profile, anti-ds DNA antibody, lupus anticoagulant (LA), anticardiolipin antibodies (ACA), IgG and IgM were recorded. LA levels 1.5 times higher than the upper limit are accepted as a high titre. ACA levels higher than 40 iU/L are accepted as a high titre. Preeclampsia, IUGR, foetal loss, preterm delivery, and the history of neonatal loss were evaluated as obstetric-perinatal outcomes. Lupus exacerbation was defined as the presence of acute arthritis, malar rash, vasculitis, serositis, psychosis, convulsions, or neurological findings other than eclampsia, leucopenia (<4000/mm3), Coombs-positive autoimmune hemolytic anaemia, or thrombocytopenia (<100,000/mm3). Renal involvement was defined as the presence of proteinuria of more than 500mg/day on at least one test, presence of dysmorphic erythrocytes, and the presence of cellular casts in urine in the absence of preeclampsia. Renal flare was defined as the presence of emerging signs of renal involvement who already have renal involvement (proteinuria of more than 500mg/day on at least one test, dysmorphic erythrocytes, and the presence of cellular casts in urine in the absence of preeclampsia).

If the pregnancy occurred after 6 months of remission and the patient was evaluated by the clinician before being pregnant, this was accepted as a planned pregnancy.

The local ethics committee has approved the study (date: 30.12.2020, number: 05).

### Statistical Analysis

Continuous data are given as mean ± standard deviation. Categorical data are given as a percentage (%). The Shapiro-Wilk test was used to investigate the suitability of the data for normal distribution. In the comparison of normally distributed groups, independent sample t-test analysis was used for cases with two groups. The Mann-Whitney U test was used for the cases with two groups in the comparison of the groups that did not conform to the normal distribution. Pearson Chi-Square and Pearson Exact Chi-Square analyses were used in the analysis of the created cross tables. IBM SPSS Statistics 21.0 (IBM Corp. Released 2012. IBM SPSS Statistics for Windows, Version 21.0. Armonk, NY: IBM Corp.) program was used in the analysis. A value of p<0.05 was accepted as a criterion for statistical significance.

## RESULTS

Fifty-five pregnancies of 31 SLE patients were included in the study. The mean age of the patients was 29.1±4.34 (26.5–32.0) and the mean disease duration was 5.91±4.13 (3.0–9.0) years. Of the patients, 30.9% (n:17) had a history of foetal loss in their previous pregnancies, and 60% (n:33) were nulliparous. Renal involvement was present in 19 pregnancies of 11 patients and the types of renal involvement were as follows; class 3 in 11 pregnancies, class 4 in 2, class 5 in 5, and class 6 in 1. Five of the patients with renal involvement had lupus flares during pregnancy and 4 of them had class 3 and 1 had class 5 lupus nephritis.

The mean week of delivery was 37.1±1.73(36.0–38.0) and the mean birth weight was 2810±573 g (2280–3300). The rate of preterm delivery was 43.3% (n:13), the incidence of IUGR was 40% (n:12), and the rate of preeclampsia was 9.09% (n:5). Spontaneous abortion was observed in 27.3% (n:15) and foetal loss in 18.2% (n:10). Maternal and neonatal death was not observed in any of the patients. The healthy birth rate was 54.5%.

Anti-SS-A was positive in 20% (n:11) of pregnant women with lupus, and anti-SS-B was positive in 3.64% (n:2). Neonatal heart block was not observed in any of the newborns. Corticosteroids, hydroxychloroquine, and azathioprine were the agents chosen for treatment during pregnancy. Only hydroxychloroquine was used in 29.1% (n:16) of the pregnancies, hydroxychloroquine, and corticosteroids in 29.1% (n:16), and hydroxychloroquine, corticosteroid, and azathioprine in combination in 18.2% (n:10). Thirteen patients (23.6%) did not receive any medication during pregnancy. The indications of azathioprine treatment during pregnancy were as follows; 8 for renal, 1 for central nervous system, and 1 for hematological involvement. Hydroxychloroquine dose was 200 mg/day for all patients. All patients receiving glucocorticoids had 20 mg or less doses of prednisolone or equivalent doses of methylprednisolone. Azathioprine doses were 100 mg/day or 150 mg/day.

A comorbid disease was present in 9 patients; 5 patients with hypothyroidism, and 4 patients with diabetes mellitus (1 type 1, 3 type 2).

The disease exacerbated during pregnancy in 5 (9.09%) cases. All exacerbations were in the form of renal flares and all had renal involvement before pregnancy. All exacerbations occurred in the 3rd trimester. The duration of disease in patients with exacerbations was 10.8±4.32 yrs, all had hypocomplementemia, and 60% (n:3) were in remission for less than six months. These patients were those who did not come for regular follow-ups. The clinical characteristics and pregnancy outcomes of the patients are summarised in **Table 1**.

**Table 1.** Clinical characteristics and pregnancy outcomes of the patients.

Age (yrs) Mean ± SD (min–max)	29.1±4.34 (26.5–32.0)
Duration of disease (yrs)	5.91±4.13
Mean ± SD (min–max)	(3.0–9.0)
Lupus anticoagulant positivity (%,(n))	36.4% (N:20)
Anticardiolipin IgG positivity (%,(n))	27.3% (N:15)
Anticardiolipin IgM positivity (%,(n))	21.8% (N:12)
Anti- SS-A positivity (%,(n))	20% (N:11)
Anti- SS-B positivity (%,(n))	3.64% (N:2)
Hypocomplementemia (%,(n))	52.7% (N:29)
Exacerbation during pregnancy (%,(n))	9.09% (N:5)
Preterm birth (%,(n))	43.3% (N:13)
Intrauterine growth retardation (%,(n))	40% (N:12)
Preeclampsia (%,(n))	9.09% (N:5)
Spontaneous abortion (%,(n))	27.3% (N:15)
Foetal loss (%,(n))	18.2% (N:10)
Healthy birth (%,(n))	54.5% (N:30)

The rate of patients with renal involvement was 34.5% (n:19). When pregnant women with and without kidney involvement were statistically compared; the exacerbation rate was 26% (n:5) vs 0% (n:0) (p:0.006), the abortion rate was 26% (n:5) vs 25% (n:9) (p:1), preterm birth rate %60 (n:6) vs 35% (n:7) (p:0.5), IUGR rate 60% (n:6) vs 30% (n:6) (p:0.35), preeclampsia rate 16% (n: 3) vs. 6% (n:2) (p:0.45), respectively. Pregnant women with kidney involvement were numerically higher in terms of abortion rate, preterm birth rate, IUGR, and preeclampsia rate, but the difference did not reach statistical significance. However, the exacerbation rate was higher in pregnant women with renal involvement between the two groups, and the difference was statistically significant (p:0.006). The comparison of patients with and without renal involvement is summarised in **Table 2**.

**Table 2.** Comparison of pregnancies with and without renal involvement.

	**With renal involvement (n:19)**	**Without renal involvement (n:36)**	**P**
Exacerbation	26 % (N:5)	0 % (N:0)	0.006
Healthy birth (%,(n))	53 % (N:10)	56 % (N:20)	1
Spontaneous abortion (%,(n))	26 % (N:5)	25 % (N:9)	1
Preterm birth (%,(n))	60 % (N:6)	35 % (N:7)	0.5
IUGR (%,(n))	60 % (N:6)	30 % (N:6)	0.35
Preeclampsia (%,(n))	16 % (N:3)	6 % (N:2)	0.45
Average week of birth	36.5 ± 1.58 (35.3 - 37.8)	37.4 ± 1.76 (36.8 - 39.0)	0.13
Average birth weight Mean ± SD (min–max)	2570 ± 700 (2050 - 3200)	2940 ± 470 (2630 - 3300)	0.13

Antiphospholipid antibody syndrome (APS) was present in 32.7% (n:18) of the cases in which pregnancies were all planned, followed up regularly, and patients used low molecular weight heparin. There was a history of foetal loss before diagnosis in 50% (n:9) of the cases with APS. LA was positive in all APS cases. LA was positive in high titre at 77.8% (n:14) and positive in low titre at 22.2% (n:4), ACA IGG was positive at 77.8% (n:14), and ACA IgM was positive at 66.7% (n:12). ACA IGG was positive in all APS patients with LA positive at high titer. The healthy birth rate of cases with APS was 33.3% (n:6), the foetal loss rate was 55.6% (n:10), preeclampsia rate was 11.1 % (n:2). Three newborns were born to pregnant women with APS had IUGR and three pregnant women with APS gave birth prematurely. The mean week of delivery was 37.0±1.26 (36.0–37.7), and the mean birth weight was 2780±563 (2240–3230) g. When the pregnant women with and without APS were compared, the foetal loss rate was 55.6% (n:10) vs 0% (n:0) (p<0.001), preterm delivery rate 50% (n:3) vs 41.66% (n:10) (p:0.61), IUGR rate was 50% (n:3) vs 41.66% (n:10) (p:0.32), preeclampsia rate was 11.1% (n:2) vs 8.11% (n:3) (p:1). In terms of preterm birth rate, IUGR rate, and preeclampsia rate, pregnant women with APS were numerically higher, but the difference did not reach statistical significance. Foetal loss was seen only in pregnant women with APS (55.6% (n:10) vs. 0% (n:0) (p<0.001)). A comparison of patients with and without APS is summarised in **Table 3**.

**Table 3.** Comparison of patients with and without APS.

	**With APS (n:18)**	**Without APS (n:37)**	**P**
Healthy birth (n,%)	6 (33.3)	24 (68.9)	0.06
Foetal loss (n,%)	10 (55.5)	0	**<0.001**
Preterm birth (n,%)	3 (16.7)	10 (27)	0.61
IUGR (n,%)	3 (16.7)	10 (27)	0.32
Preeclampsia (n,%)	2 (11.1)	33 (89.2)	1
Average week of birth	37.0±1.26 (36.0–37.7)	37.1±1.285 (36.3–38.0)	0.71
Average birth weight Mean ± SD (min–max)	2780±563 (2240–3230)	2940±649 (2830–3300)	0.5

Six (10.9%) of the 55 pregnancies in our study were unplanned pregnancies. Of these six pregnancies, five resulted in abortion and only one resulted in a healthy birth at 35 weeks of gestation, and IUGR was detected in the foetus. Exacerbation was observed in 3 of the unplanned pregnancies. When planned and unplanned pregnancies were compared statistically; the exacerbation rate was 4% (n:2) vs 50% (n:3) (p:0.003), the abortion rate was 20% (n:10) vs 83% (n:5) (p:0.05), preterm birth was rate %41.3 (n:12) vs 100% (n:1) (p:1), IUGR rate was 37.9% (n:11) vs 100% (n:1) (p:1), preeclampsia rate was 10% (n: 5) vs 100% (n:0) (p:0.95). Unplanned pregnancies were numerically higher in terms of abortion, preterm birth, IUGR, and preeclampsia, but the difference did not reach statistical significance. However, the exacerbation rate was higher in unplanned pregnancies between the two groups, and the difference was statistically significant (50% vs 4% (p:0.003)).

Patients with renal flares are in remission with immunosuppressive treatment.

## DISCUSSION

SLE, which is common in women of childbearing age, is associated with obstetric complications such as spontaneous abortion, premature birth, intrauterine growth retardation, foetal loss, preeclampsia, increased maternal morbidity, and mortality.^2^ In the current study, there were 55 pregnancies in a total of 31 SLE patients. The foetal loss occurred in 30.9% of the patients, preterm delivery in 43.3%, IUGR in 40%, and preeclampsia in 9.09%. Spontaneous abortion was seen in 27.3% of the patients. There was no history of neonatal loss, neonatal heart block, or maternal death. The healthy birth rate was 54.5%. SLE exacerbation was observed in 9.09% of the cases, and all of these patients had renal involvement before pregnancy, and 60% had a pre-pregnancy remission period of fewer than 6 months.

The exacerbation rate of SLE during pregnancy was found to be 9.09% in the current study. In previous studies, the rate of exacerbation of the disease during pregnancy was reported as 13–33%.^13^ The reason for the lower exacerbation rate in here may be that the pregnancy of 89.1% (n: 49) of the patients occurred after a state of remission lasting longer than 6 months. It has been shown that the risk of exacerbation is lower if the disease is in remission at the time of conception, whereas the risk of exacerbation is higher in those with active disease at conception or before pregnancy.^11,14^ Since most of the pregnant women in our study were followed by a rheumatologist and given pre-pregnancy counseling, exacerbation rates were not high. In the current study, exacerbation occurred during pregnancy in 5 (9.09%) of 55 pregnancies. All of these exacerbations were in the form of renal involvement. It was observed that 60% of these pregnant women who had an exacerbation of SLE during their pregnancy had active lupus nephritis at conception or in the 6 months before pregnancy. Active lupus nephritis is a contraindication for pregnancy because it is associated with both maternal and foetal complications leading to severe anuric renal failure and even maternal death.^15^

Abortion rates in pregnancies of SLE patients are higher (6–35%) than in the general population.^16^ Consistent with the literature, 27.3% of the pregnancies ended in miscarriage. Antiphospholipid antibody positivity was reported as 21% and 17% in 2 studies conducted in pregnant SLE cases.^17,18^ APS may cause placental insufficiency, which may result in foetal loss, and requires anticoagulant therapy. In our study, this rate was 36.4%. The reason for higher antiphospholipid antibody positivity than in the literature may be that all patients diagnosed with SLE were routinely screened for secondary APS. Previously reported live birth rates in the pregnant population with SLE were reported as 85% in a retrospective study conducted with 72 pregnant women, and 85.6% in a similar prospective study.^19,20^ In the current study, the live birth rate was found to be 54.5%. The reason for the slightly lower live birth rate may be the high rates of antiphospholipid antibody positivity in the study population when compared with the previous studies. In a previous study conducted on pregnant women with SLE, the rate of foetal loss was reported as 0–22%.^21^ Herein, the rate of foetal loss was found to be 18.2%, similar to the literature. The high foetal loss rate may also be the result of the high rate of pregnant women with lupus who have positive antiphospholipid antibodies. Because all of the pregnant women with foetal loss had APS. It has been reported in the literature that the incidence of preeclampsia is increased in lupus patients, and the incidence varies between 5 and 38%.^17,21^ We detected the incidence of preeclampsia as 9.09% in line with the literature. It is known that APS is an important cause of preeclampsia^18^ and the incidence of preeclampsia was higher in patients with APS (11.1% vs 8.11%) in our study.

Even though we have some striking results, our study has some limitations. One of them is the small number of patients for making inferences. However considering the frequency of SLE in the general population and compared with the published data, we have a respectable number of patients for a single center. Another limitation is the retrospective design of our study which may have caused a loss of data. Finally, we do not have a control group consisting of healthy subjects or pregnant having some other type of inflammatory disorder to compare. In conclusion, it was observed that the rates of exacerbation, abortion, premature birth, intrauterine growth retardation, and preeclampsia in planned pregnancies were significantly lower than in unplanned pregnancies. Exacerbations are minimal in patients with well-controlled disease. Since SLE can be seen in women of childbearing age and normal fertility, patients should be warned about planned pregnancy. As it is known, APS is the most important cause of pregnancy complications such as preterm birth, foetal loss, and preeclampsia. For this reason, every SLE patient planning a pregnancy should be questioned in terms of APS, and examined, and her pregnancy should be followed closely. Therefore, pre-pregnancy counseling and ensuring that they get pregnant under appropriate conditions are extremely important in terms of obtaining positive obstetric outcomes. However, it seems that it is possible to achieve satisfactory results thanks to the cooperation of the patient, rheumatologist, and obstetrician.
